# Shoulder muscle changes in patients with type 2 diabetes mellitus who have a painful shoulder: a quantitative muscle ultrasound study

**DOI:** 10.1186/s12891-022-05627-9

**Published:** 2022-07-14

**Authors:** Login Ahmed S. Alabdali, Bjorn Winkens, Geert-Jan Dinant, Nens van Alfen, Ramon P. G. Ottenheijm

**Affiliations:** 1 Dept. of Family Medicine, CAPHRI Care and Public Health Research Institute, Maastricht University, Maastricht, The Netherlands; 2 Ministry of Education, Riyadh, Kingdom of Saudi Arabia; 3 Department of Methodology and Statistics, CAPHRI Care and Public Health Research Institute, Maastricht University, Maastricht, the Netherlands; 4 Dept. of Neurology and Clinical Neurophysiology, Radboud University Medical Center, Donders Institute for Brain Cognition and Behaviour, Nijmegen, The Netherlands

**Keywords:** Echogenicity, Muscle denervation, Quantitative muscle ultrasound, Shoulder pain, Type 2 diabetes mellitus

## Abstract

**Background:**

It is assumed that in patients with diabetic neuropathy, muscle denervation can result in shoulder disorders. Muscle denervation will lead to changes in muscle architecture, which can be assessed by quantitative muscle ultrasound (QMUS). The aim was to investigate whether increased muscle echogenicity, as a sign of neuropathy, is more often present in patients with shoulder pain who have type 2 diabetes mellitus (T2DM) than in those without.

**Methods:**

Sixty-six patients with T2DM and 23 patients without diabetes mellitus (DM) having shoulder pain were included. Quantitative muscle ultrasound images were obtained bilaterally from the biceps brachii, deltoid, and supra- and infraspinatus muscles. The mean echogenicity (muscle ultrasound grey value) was transformed into z-scores and compared to reference values obtained from 50 healthy participants. Associations between muscle echogenicity and clinical variables were explored.

**Results:**

In painful shoulders of both patients with T2DM and patients without DM, mean echogenicity z-scores of all muscles were significantly increased compared to healthy controls. No significant differences in echogenicity between patients with T2DM and those without DM were found. In patients with T2DM, a distal symmetric polyneuropathy was significantly associated with increased echogenicity of all muscles except the infraspinatus muscle.

**Conclusions:**

These findings indicate that patients with painful shoulders, irrespective of having T2DM, seem to have abnormal shoulder muscles. Future studies are needed to elucidate whether neuropathy or other conditions lead to these muscle changes.

**Supplementary Information:**

The online version contains supplementary material available at 10.1186/s12891-022-05627-9.

## Introduction

Diabetes mellitus (DM) is associated with shoulder disorders such as adhesive capsulitis and rotator cuff disorders [1, 2]. These disorders are primarily regarded as connective tissue disorders, but neuropathy is also hypothesized to be the cause of increased incidence of these musculoskeletal disorders in patients with DM [3]. It is known that diabetic neuropathy increases the risk of developing adhesive capsulitis of the shoulder [4]. Currently, shoulder management is not aimed at neuropathic disorders [1], while it is shown that patients with a subacromial pain syndrome might suffer from sensitization and not only nociceptive pain [5, 6]. Diabetic neuropathy is the most prevalent complication of DM, that affects approximately 50% of all patients [7]. This heterogeneous group of conditions affects somatic and autonomic nerves and presents with diverse clinical forms [8]. The American Diabetic Association advises to classify neuropathy in patients with DM as either diffuse neuropathy (e.g. distal symmetric polyneuropathy), mononeuropathy or (poly) radiculopathy [8]. Distal symmetric polyneuropathy is the most studied and prevalent presentation in patients with DM [8]. Diabetic radiculoplexus neuropathy, also known as diabetic amyotrophy, has been mainly observed in the proximal thigh nerves (femoral, sciatic, and obturator nerves and lumbosacral plexus) [9], and those patients have typically type 2diabetes mellitus (T2DM) [10]. Shoulder neuropathies in patients with DM has been described, but are currently regarded as rare [11–15]. A systematic study of the occurrence of shoulder neuropathies in patients with DM suffering from shoulder pain, showed that neuropathic shoulder pain was present in 3% of the patients with T2DM [15]. However, results from this study were only based on the history and physical examination.

It has been postulated that in patients with diabetic neuropathy muscle denervation results in shoulder disorders [9]. Muscle denervation will lead to changes in muscle architecture as healthy muscle fibers become atrophied and muscle tissue is replaced by fat and fibrosis [16]. These muscle changes can be observed by ultrasound imaging (US) [17]. US will then show a brighter appearance, or increased echogenicity, of the muscle on the screen. Quantitative muscle ultrasound (QMUS) is a reliable and patient-friendly technique to assess changes in echogenicity. These changes, as a result of denervation, have been studied successfully with QMUS [17]. Quantification of muscle echogenicity using greyscale analysis has a high inter-observer agreement than visual evaluation, with good clinical validity for detecting neuromuscular pathology [18]. Greyscale analysis requires minimal training and increases diagnostic sensitivity to 92% compared to visual image grading [17, 19]. It can be used as a screening tool for the presence of neuromuscular disorders [20], and is currently regarded as the most sensitive and reliable ultrasound measure used for detecting such disorders [19]. QMUS allows for echogenicity comparison with healthy reference values, which are corrected for sex, age and weight.

Increased muscle echogenicity can also be caused by other factors than muscle denervation. In patients with DM, higher levels of intramuscular adipose tissue can be found compared to people without DM [21], which increases echogenicity [22]. In addition, full-thickness tears of the supra- or infraspinatus tendon can also lead to fatty degeneration of the affected muscle [23].

Our aim was to investigate whether increased muscle echogenicity, as a possible sign of diabetic neuropathy, is more often present in patients suffering from shoulder pain who have T2DM, using QMUS. This association would provide new information that may help to better direct disease management and preventing shoulder joint complications in T2DM.

## Methods

### Study population and study setting

All patients with T2DM suffering from shoulder pain who were enrolled in a questionnaire study in general practice, assessing the prevalence of musculoskeletal disorders in patients with T2DM, were eligible for this cross-sectional study and were invited to this study [15]. Since insulin resistance is positively associated with increased intermuscular adipose tissue in muscles [21], and we did not want to include two possible etiological factors for increased echogenicity, patients without DM suffering from shoulder pain were also included in this study. Patients without DM were recruited during their visit to a diagnostic center for shoulder ultrasound imaging after referral by their general practitioner. The inclusion criteria for patients with T2DM or without DM were: age between 30 and 70 years, shoulder pain that had lasted longer than 4 weeks and was not caused by trauma. To determine the reference values for echogenicity, we also aimed to examine 50 healthy participants within the age and BMI range of the patient population, and with the same sex ratio as the patients with T2DM suffering from shoulder pain. Healthy subjects were recruited among colleagues of the researchers.

Patients and healthy participants were excluded if they had a history of self-reported (poly-)neuropathy other than a diabetic distal symmetric polyneuropathy, or a myopathy. All patients gave written informed consent and were asked for their permission to send a letter to their general practitioners with the positive findings of the physical and shoulder musculoskeletal ultrasound examination. The study was approved by the Medical Ethics Committee of Zuyderland Medical Centre (METC-Z 17-T-138).

### Demographic and clinical characteristics

Age, sex, height, weight, hand dominance, and HbA1C for patients with T2DM were obtained at inclusion, and the body mass index (BMI) was calculated. The 10-item Douleur Neuropathique questionnaire (DN4) was performed to assess the presence of neuropathic shoulder pain. The DN4 is a commonly used questionnaire that includes a physical examination for screening and diagnosing neuropathic pain in patients with neurological complaints. It has been validated for the Dutch population and in DM patients [24–26]. We used a cut-off score for neuropathic pain of ≥5 out of 10 points [24]. The shoulder was inspected for atrophy of the deltoid, supraspinatus and infraspinatus muscles, and for winging of the scapula as a possible sign of a concomitant neuralgic amyotrophy. Neuralgic amyotrophy, also known as idiopathic brachial plexus neuropathy, and typically involving the long thoracic, suprascapular, and anterior interosseous nerves, causes muscle denervation [27]. It is important to notice that no association between DM and neuralgic amyotrophy has been verified [27]. Cervical spine flexion, extension, rotation and lateral flexion, and a combination of rotation and extension were tested to see if they caused radiating pain at the affected side as a possible sign of cervical radiculopathy.

All patients with shoulder pain underwent a neurological examination of the feet and shoulders as previously described [15]. Shoulders were examined according to the shoulder pain guidelines of the Dutch College of General Practitioners [28]. Physical examination findings were used to diagnose the following disorders: subacromial pain syndrome (SAPS), a glenohumeral or an “other disorder”, and categorized using a mutually exclusive method leading to a single diagnosis. Glenohumeral disorders were defined by an external rotation range of motion of less than 45 degrees, while SAPS was defined by either a painful abduction (including a painful arc) with or without a limited range of motion during abduction, or a positive Hawkins–Kennedy and Neer test. An “other disorder” was defined as not having any of the two previous disorders, and includes for example AC-joint disorders [15]. Examination of lower extremity symptoms and signs included an interview part to assess signs of distal symmetric polyneuropathy (DSP) and a physical exam with inspection for atrophy, strength testing to assess distal muscle weakness, ankle reflexes, and sensory testing of the vibration sense with a128-Hz Rydel-Seiffer tuning fork, and touch sensation with a Semmens-Weinstein monofilament. Criteria for the diagnosis of distal symmetric polyneuropathy were as previously described, and based on a combination of signs and symptoms [15, 29].

In all patients their hands were examined for the presence of a “prayer sign” or positive “tabletop sign” as a manifestation of a so-called stiff hand syndrome, one of the frequently observed upper extremity complications of DM [30].

### Shoulder musculoskeletal ultrasound

We performed conventional shoulder US following the guidelines of the European Society of Musculoskeletal (MSK) Radiology [31, 32], with specific attention to the supra- and infraspinatus rotator cuff tendons to check for full thickness tears that can lead to fatty degeneration of the affected muscle [23]. For this, we used a Phillips EPIQ 7G ultrasound machine (Philips Healthcare, Eindhoven, The Netherlands) and a broadband 4–18 MHz linear transducer, using the higher frequencies (“2D Opt resolution”) and the shoulder MSK preset. Scanning was performed by one of three experienced radiologists. A full-thickness tendon tear was diagnosed in the presence of any of the following criteria: focal tendon thinning, complete tendon non-visualization, focal tendon discontinuity with homogeneous echogenicity without focal thinning, or inversion of the superficial bursa contour and/or hyperechoic tissue [32].

### Quantitative muscle ultrasound

Using a standardized protocol, ultrasound imaging of the following four muscles was performed with a linear 5–10 MHz transducer (Siemens Acuson P500, Siemens Healthcare, Germany), of the supra- and infraspinatus, deltoid, and biceps brachii muscles bilaterally. As the type of ultrasound device influences echogenicity values, the exact same device and a fixed preset settings were used throughout the study for all participants (MSK preset, gain 1 dB, dynamic range compression of 80, one focal zone at a fixed image depth of 5 cm). All patients were seated on a chair with the shoulders in neutral position and arms relaxed and the elbow flexed, resting on a pillow with the palm faced up. Three images were captured of each muscle studied, removing the probe between measurements, and ensuring an optimal perpendicular angle to a predefined underlying reflective structure (bone or fascia) in the image, with a generous amount of gel to avoid pressure on the skin. Images were stored digitally for further analysis. All muscle imaging was performed by one of the authors (LA).

**Fig. 1:**
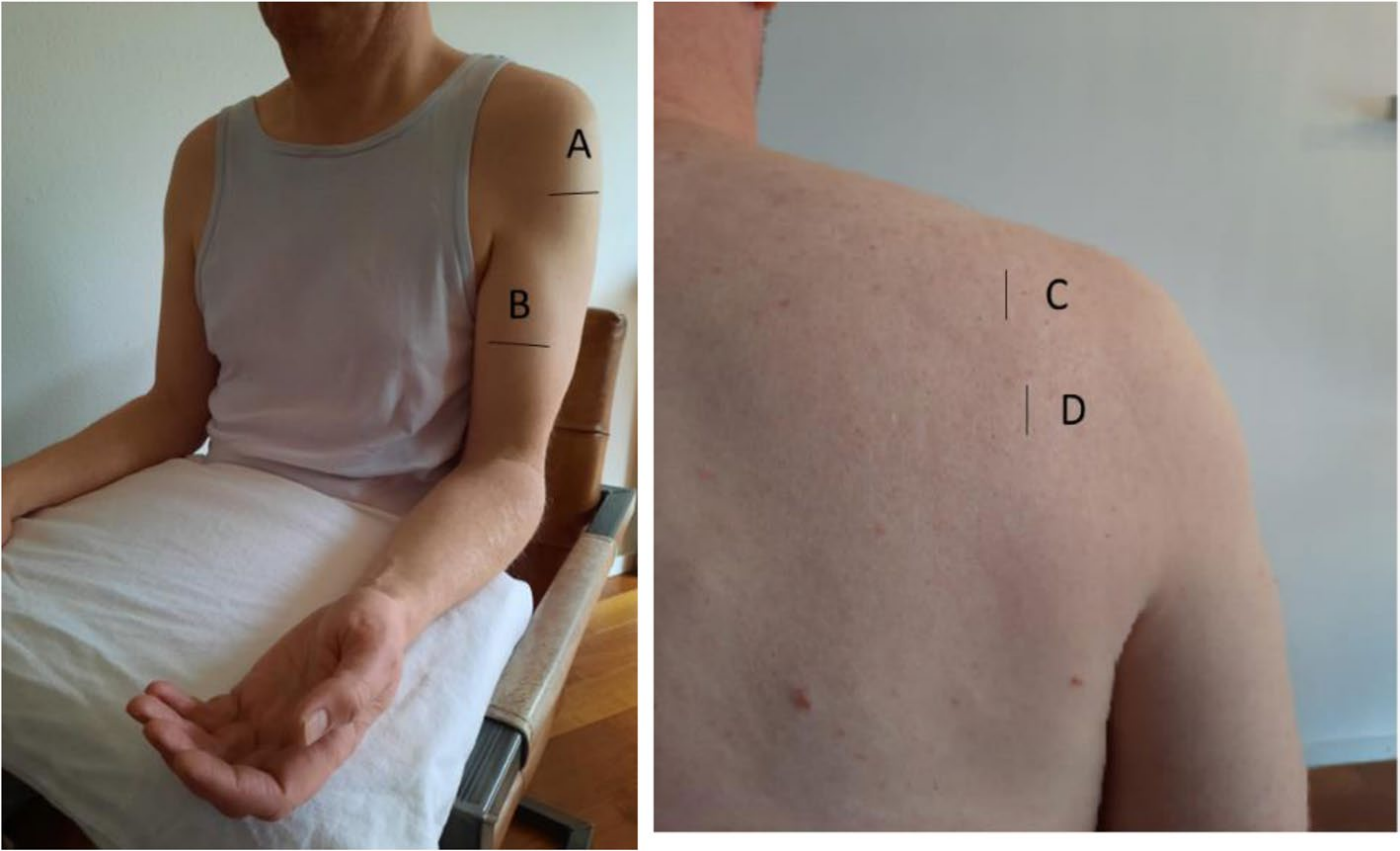
Scanning locations of the four investigated muscles for quantitative muscle ultrasound. **A**, location for deltoid muscle; **B**, location for biceps brachii; **C**, location for supraspinatus muscle; **D**, location for infraspinatus muscle

All images were made in the transverse plane at a standard transducer location (Fig. 1). The supraspinatus muscle was scanned with the transducer placed at one-fourth along the line from the lateral acromion to the vertebral midline. For the infraspinatus muscle, the transducer was moved caudally from the previous position, passing over the scapular spine. The middle deltoid muscle was scanned with the transducer placed at one-fourth of the distance from the acromion to the lateral epicondyle keeping the forearm in relaxed supination. For the biceps brachii muscle, the transducer was placed at two-thirds of the distance from acromion to the antecubital crease on the ventral upper arm.

### Measurement of muscle echogenicity

All images were assessed offline by manually selecting a region of interest (ROI) in each image, to obtain the average greyscale level using the histogram function of ImageJ software (version 1, Madison, W.I., USA; https://imagej.nih.gov), where black = 0 and white = 255. For each patient, the same area was chosen as a region of interest. As the outlines of the muscle can be difficult to determine in case of severe neuromuscular pathology, the underlying bone or interosseous membrane was chosen as reference point for depth measurements in each image. The results of the three measurements per muscle were averaged to optimize reliability and decrease the variation [33].

### Muscle echogenicity reference values

The averaged echogenicity per muscle was also calculated for the healthy participants who were recruited in same age and BMI range of patients suffering from shoulder pain. As sex, age, BMI and hand dominance can affect echogenicity of muscles [33], multiple linear regression analyses were performed separately for females and males, and per side to assess how the covariates age, BMI, and dominant hand affected the echogenicity of the four muscles. After applying a backward stepwise elimination method (significance level α = 0.05) [22], the variables that were statistically significantly associated with echogenicity remained in the model, which was then used to compute the reference values for the patients with shoulder pain [33]. The general regression formula was:

$$\mathrm{Echogenicity}\;=\;\mathrm C\;+\;(\mathrm\alpha\cdot\mathrm{age}-\mathrm{centered})\;+\;(\mathrm\beta\cdot\mathrm{age}-\mathrm{centered}^2)\;+\;(\mathrm\gamma\cdot\mathrm{age}-\mathrm{centered}^3)\;+\;(\mathrm\delta\cdot\mathrm{BMI})\;+\;(\mathrm\zeta\cdot\mathrm{dominant}\;\mathrm{hand}).$$

Note that if a variable was not significantly related to echogenicity, then it was removed from the model, which means that its regression coefficient (α, β, γ, δ, or ζ) in this formula is set to equal 0. If no variable was significant, then the mean echogenicity of the healthy participants and their corresponding standard deviation (SD) was reported.

### Muscle echogenicity in all patients suffering from shoulder pain

The averaged echogenicity per muscle of a person was transformed into a z-score using the reference values and the residual standard deviation (SD) obtained from the regression analysis [34]:

$$\mathrm{Echogenicity}\;\mathrm z-\mathrm{score}\;=\;(\mathrm{measured}\;\mathrm{value}\;-\;\mathrm{reference}\;\mathrm{value})\;/\;\mathrm{SD}.$$

### Statistical analysis

Numerical variables were described using the mean and standard deviation (SD). Numbers with percentages of shoulders or patients were used for categorical variables. For baseline characteristics, comparisons between the three study groups (healthy participants, patients with T2DM and without) were performed using ANOVA or chi-square test where applicable. The mean echogenicity z-values of painful shoulders in patients with T2DM and those without DM suffering from shoulder pain were compared to the reference values; i.e. comparing it with z = 0, and with each other using a marginal model for repeated measures with group (two categories, i.e. patients suffering from shoulder pain with and without DM) as a fixed factor, and an unstructured covariance structure to account for clustering of painful shoulders within a patient suffering from bilateral pain. Shoulders are considered biologically related, therefore, parametric and non-parametric tests for comparing two groups were not appropriate for this analysis.

The difference in mean echogenicity z-scores between painful and non-painful shoulders within patients with T2DM was analyzed using marginal model for repeated measures with painful shoulder (yes/no) as fixed factor and an unstructured covariance structure.

Next, the frequency of abnormal echogenicity z-scores per muscle in painful shoulders of patients with shoulder pain was calculated, with z-scores exceeding ≥2.0 considered abnormal for individual muscles. The difference in frequency of abnormal echogenicity z-scores of painful shoulders between groups was assessed using generalized estimating equation with an unstructured working correlation matrix structure and group (three categories, i.e. patients suffering from shoulder pain with and without DM and healthy participants) as fixed factor.

Finally, linear regression analyses were performed in both groups of patients with T2DM and patients without DM separately, to investigate the association between the echogenicity z-scores of one of the four muscles of painful shoulders (dependent variable) and the following clinical variables: duration of pain, the presence of a distal symmetric polyneuropathy, scapular winging, signs of a cervical radiculopathy, stiff hand syndrome, a supraspinatus tendon tear (only included for echogenicity of supraspinatus), and for patients with T2DM also the HbA1C level and the duration of their DM. These regression analyses were performed on a patient level, using an average z-score for the left and right shoulder for patients with bilateral shoulder pain, since the clinical variables were also measured on the patient, and not shoulder, level. Multicollinearity was checked using variance inflaction factors (VIF), where VIF > 10 indicate a collinearity problem, and influential outliers were defined as Cook’s distance > 1. Linearity assumption for numerical variables was assessed using plots. Due to a limited number of patients without DM, a selection of independent variables was made based on clinical importance and prevalence.

Statistical analyses were performed using IBM SPSS Statistics for Windows (version 25.0, Armonk, N.Y., USA). Two-sided *p-*values ≤0.05 were considered statistically significant.

## Results

### Demographic and clinical characteristics of participants

Fifty healthy participants were included in the study (30 females, mean age 50.0 years, and mean BMI 23.8). Sixty-six patients with T2DM suffering from shoulder pain (mean age 63.0 years and mean BMI 28.5), and 23 patients with shoulder pain but without DM (mean age 54.5 and mean BMI 27.5) were included in this study. Bilateral shoulder pain was present in 40% (*n =* 27) of the patients with T2DM and in 13.0% (*n =* 3) of the patients without DM. Stiff hand syndrome was present in 42.4% (*n =* 28) of the patients with T2DM, and in 17.3% (*n =* 4) without DM. Neuropathic shoulder pain was only present in 3% (*n =* 2) of the patients with T2DM. A distal symmetric polyneuropathy was found in 56.0% (*n =* 37) of the patients with T2DM, and in a similar 56.5% (*n =* 13) of the patients without DM. SAPS was in the T2DM group as well as in the patients without DM group the most common shoulder diagnosis (resp. 61% (*n =* 58) and 69% (*n =* 18)), followed by GH-disorders (resp. 18% (*n =* 17) and 23% (*n =* 6)), and other disorders (resp. 20% (*n =* 18) and 0.1% (*n =* 2)). A full-thickness tendon tear of the supraspinatus muscle was only seen in patients with T2DM, in 12.0% (*n =* 8). Clinical cervical radiculopathy was present in 13.6% (*n =* 9) of the patients with T2DM and in a similar 13.0% (*n =* 3) of the patients without DM. Winging scapula was present in both patient groups with almost equal percentages, 12.1% (*n =* 8) in patients with T2DM and 13% (*n =* 3) in patients without DM.

Group characteristics are presented in Table 1, with a detailed overview of the neurological examination in Table S1 of the Supplementary data.

**Table 1:** Demographic and clinical variables of patients with type 2 diabetes mellitus and without diabetes mellitus suffering from shoulder pain and healthy participants

	Patients with shoulder pain		Healthy participants ***n =*** 50	Comparison between groups ***P-***value
	Type 2 diabetes mellitus ***n =*** 66	Without diabetes mellitus ***n =*** 23		
Age				< 0.001^1^
mean, SD	63.0 ± 6.9	54.5 ± 9.2	50.0 ± 11.0	
range (years)	38–70	35–69	31–67	
Female sex	19 (28.8)	15 (65.2)	30 (60.0)	< 0.001^2^
BMI (kg/m^2^)				< 0.001^3^
mean ± SD	28.5 ± 4.2	27.5 ± 3.5	23.8 ± 2.9	
range	16–41.5	21–36	18.1–31.5	
Dominant shoulder affected	26 (39.3)	8 (34.7)	NA	0.001
Bilateral shoulder pain	27 (40.9)	3 (13.0)	NA	0.020
Duration of diabetes mellitus				NA
mean ± SD	9.0 ± 5.5	NA	NA	
range (years)	1–27			
Hemoglobin A1c (mmol/mol)				NA
mean ± SD	55.7 ± 7.7	NA	NA	
range	43–75			
Stiff hand syndrome	28 (42.4)	4 (17.3)	NA	0.043
Signs of cervical radiculopathy	9 (13.6)	3 (13.0)	NA	0.626
Signs of shoulder neuropathy				
DN4 score ≥ 5	2 (3.0)	0	NA	1.000
Winging scapula	8 (12.1)	3 (13.0)		1.000
Muscle atrophy	2 (6.0)*	0		1.000
DSP^#^				
Clinical	19 (28.8)	4 (17.4)	NA	0.408
Subclinical	18 (27.3)	9 (39.1)		0.309
Shoulder diagnosis				
SAPS	58 (61.2)^§^	18 (69.2)^§§^		0.519
Glenohumeral disorders	17 (18.2)^§^	6 (23.1)^§§^	NA	0.584
Other (e.g. ACJ-disorders)	18 (20.4)^§^	2 (0.1)^§§^		0.237
Full-thickness tendon tear				
Supraspinatus	8 (12.1)	0	NA	0.058
Infraspinatus	0	0		

Values are presented as absolute number and percentage unless otherwise stated; SD: standard deviation; DN4: Douleur Neuropathique 4 questionnaire; DSP: distal symmetric polyneuropathy; SAPS: Subacromial Pain Syndrome; ACJ: Acromioclavicular; * atrophy of the following muscles was observed: deltoid (*n =* 1, 1.5%), supraspinatus (*n =* 2, 3.0%), infraspinatus (*n =* 1, 1.5%); # Detailed overview of all DSP results are presented in Table S1 (supplementary data); §:based on 93 symptomatic shoulders; §§:based on 26 symptomatic shoulders; ^1^*P-*value of ANOVA test, in detail: comparison between patients with T2DM and healthy participants, *p-*value < 0.0001: between patients with T2DM and without diabetes mellitus, *p-*value = 0.004: between patients without diabetes mellitus and healthy participants, *p* value = 0.048; ^2^*P-*value of Fisher’s Exact test; ^3^*P-*value of ANOVA test, in detail: comparison between patients with T2DM and healthy participants, *p-* value < 0.0001: between patients with T2DM and without diabetes mellitus, *p-*value = 0.257: between patients without diabetes mellitus and healthy participants, *p* value< 0.0001

### Echogenicity

The regression formulas of the four muscles from the 50 healthy participants are provided in Table S2 of the Supplementary data.

**Fig. 2:**
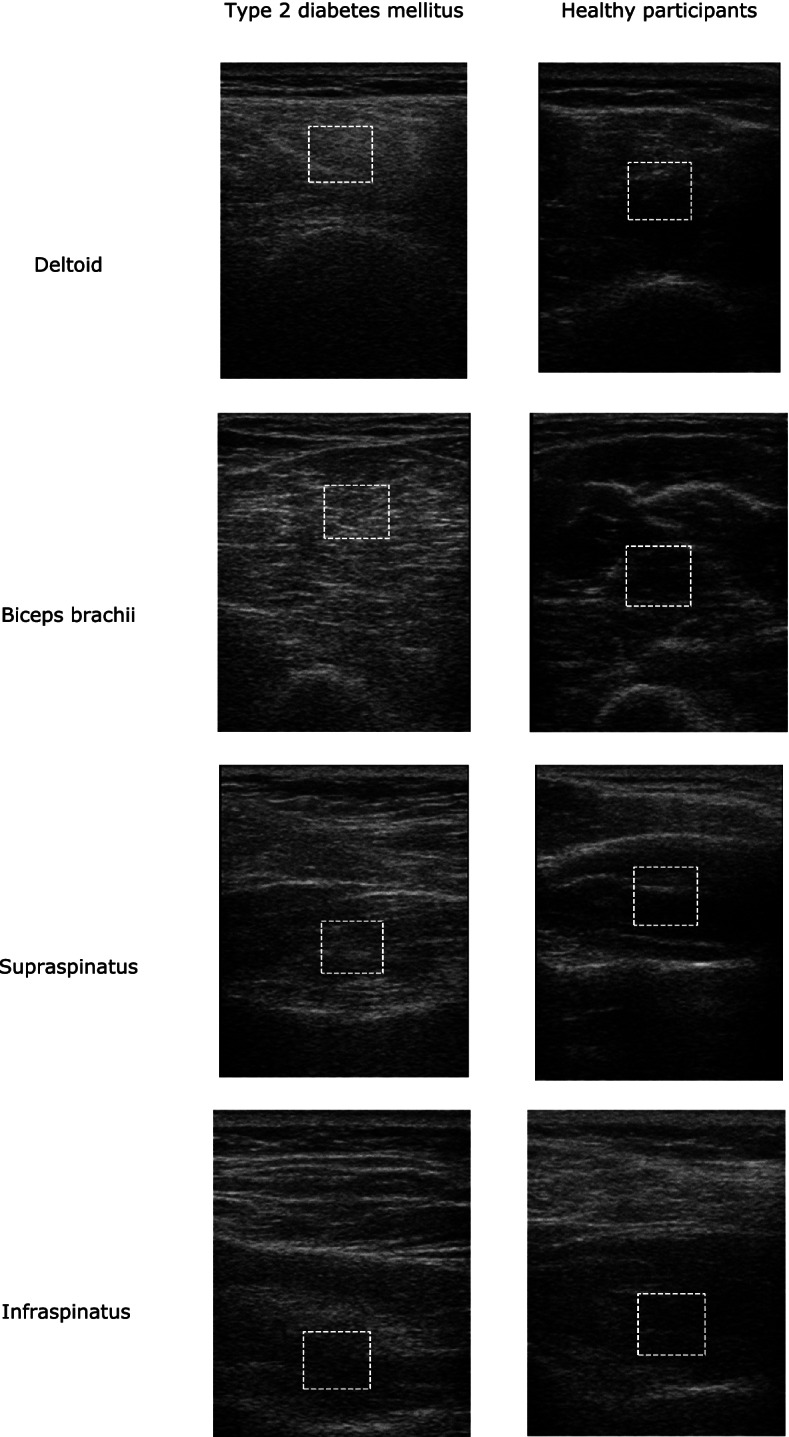
Muscle ultrasound images of patients with type 2 diabetes mellitus having shoulder pain and healthy participants. Compared with normal muscles, patients with type 2 diabetes mellitus have muscles characterized by a higher echogenicity (whiter appearance). The white dashed lines delineating the region of interest were manually chosen for analysis

In painful shoulders of both patients with T2DM and patients without, the mean echogenicity of all four muscles was significantly higher than the reference values (Table 2). Figure 2 depicts the US images of the four muscles in a healthy participant and a patient with T2DM suffering from shoulder pain. We observed that the mean echogenicity of the supraspinatus, infraspinatus and biceps brachii muscles tended to be higher in patients with T2DM compared to those without DM, but these differences were not statistically significant for any muscle (all *p-*values ≥0.201). Additionally, in patients with T2DM, the mean echogenicity did not significantly differ between painful and non-painful shoulders (results not shown, all *p-*values ≥0.231).

**Table 2:** Differences in mean echogenicity (expressed in z-score) of painful shoulders in patients with type 2 diabetes mellitus and patients without diabetes mellitus from reference values^a^

	Painful shoulders					
	Type 2 diabetes mellitus ***n =*** 93			Without diabetes mellitus ***n =*** 26		
Z-score	Mean	95% CI	*P-*value	Mean	95% CI	*P-*value
Supraspinatus	0.8	0.4, 1.1	< 0.001	0.6	0.2, 0.9	0.009
Infraspinatus	1.2	0.6, 1.6	< 0.001	0.9	0.3, 1.4	0.006
Deltoid	1.0	0.5, 1.5	< 0.001	1.0	0.3, 1.7	0.006
Biceps brachii	1.1	0.7, 1.5	< 0.001	1.0	0.4, 1.7	0.004

^a^ no significant difference between patients with and without type 2 diabetes mellitus was found (all *p-*values ≥0.201)

The total number of shoulders with at least one muscle that had a z-score ≥ 2 in patients with T2DM was 60 (64.5%), and 18 in patients without DM (69.2%). In patients with T2DM, 113 of 372 (27 patients had bilateral pain) muscles on the painful side (30.4%) had a z-score of ≥2, while in patients with shoulder pain without DM there were 23 muscles (22.0%, 23/104 (3 patients had bilateral pain)) with a z-score ≥ 2 (Table 3).

**Table 3:** Frequency of abnormal z-scores (≥ 2.0) of echogenicity values of painful shoulders in patients with type 2 diabetes mellitus and patients without diabetes mellitus

	Patients with type 2 diabetes mellitus		Patients without diabetes mellitus		Comparison between both patient groups
Z-score ≥ 2.0	n (%)	95% CI	n (%)	95% CI	*P-*value
Supraspinatus	17 (18.3)^#^	0.1, 0.3	4 (15.4)^#^	0.1, 0.4	0.374
Infraspinatus	33 (35.5) ^*^	0.3, 0.5	5 (19.2) ^*^	0.1, 0.4	0.126
Deltoid	30 (32.3) ^*^	0.2, 0.4	7 (26.9) ^*^	0.1, 0.5	0.667
Biceps brachii	33 (35.5) ^*^	0.3, 0.5	7 (26.9) ^*^	0.1, 0.5	0.443

* *P-*value < 0.01 when compared to reference (healthy participants)

# *P-*value < 0.05 when compared to reference (healthy participants)

Of the muscles with a z-score ≥ 2 in painful shoulders in patients with T2DM, the highest frequency of abnormalities was observed in the infraspinatus and biceps brachii muscles (35.5%, 33/93), followed by the deltoid muscle (32.3%, 30/93), and supraspinatus muscle (18.3%, 17/93). In patients with shoulder pain but without DM, the abnormalities were most often found in the deltoid and biceps brachii muscles (26.9%, 7/26), followed by infraspinatus (19.2%, 5/26) and supraspinatus muscles (15.4%, 4/26). Differences between patients with T2DM and those without DM were not statistically significant (Table 3).

### Associations between echogenicity and clinical variables

The associations in patients with T2DM between echogenicity and the clinical variables are presented in Table 4; only the presence of a distal symmetric polyneuropathy was significantly associated with echogenicity z-scores of all muscles, except for the infraspinatus muscle. More specifically, the z-score echogenicity was on average higher for patients with a distal symmetric polyneuropathy than in those without: in the supraspinatus muscle the average z-score was 0.93 (95%CI: 0.08, 1.77, *p* = 0.032), in the deltoid it was 1.50 (95%CI: 0.45, 2.55; *p* = 0.006), and in the biceps brachii 1.33 (95% CI: 0.44, 2.21, *p* = 0.004). The results for patients without DM are presented in Table 5; no significant associations were found.

**Table 4:** Associations between the echogenicity of the supraspinatus, infraspinatus, deltoid and biceps brachii muscle of the painful shoulders (expressed in z-scores) and clinical variables in patients with type 2 diabetes mellitus suffering from shoulder pain (*n =* 66)

Clinical variables	Muscles											
	Supraspinatus			Infraspinatus			Deltoid			Biceps		
	B	95% CI	*P-*value	B	95% CI	*P-*value	B	95% CI	*P-*value	B	95% CI	*P-*value
Hemoglobin A1c	0.02	−0.03, 0.1	0.407	− 0.01	− 0.1, 0.1	0.826	0.03	− 0.04, 0.1	0.372	0.01	−0.04, 0.1	0.616
Duration of diabetes mellitus	−0.03	−0.1, 0.04	0.442	−0.02	− 0.1, 0.1	0.683	− 0.09	−0.2, 0.01	0.072	0.003	−0.1, 0.1	0.941
DSP	0.9	0.1, 1.7	0.032	1.001	−0.1, 2.1	0.069	1.5	0.5, 2.6	0.006	1.3	0.4, 2.2	0.004
Signs of shoulder neuropathy	−0.1	−1.2, 0.9	0.798	0.04	− 1.3, 1.4	0.952	0.1	−1.2, 1.4	0.848	−0.8	−1.9, 0.3	0.165
Signs of cervical radiculopathy	−0.02	−1.2, 1.2	0.977	0.3	−1.2, 1.9	0.685	0.8	−0.7, 2.3	0.293	−0.1	−1.3, 1.2	0.931
Stiff hand syndrome	0.4	−0.5, 1.2	0.430	0.3	−0.9, 1.4	0.660	0.8	−0.3, 1.9	0.158	0.3	−0.7, 1.2	0.557
Duration of pain	−0.003	−0.01, 0.1	0.422	−0.001	− 0.01, 0.01	0.884	0.002	−0.01, 0.01	0.633	0.003	−0.01, 0.01	0.454
SSP full-thickness tear	1.2	−0.5, 3.0	0.166	NA								

B Multiple level linear regression coefficient, *DSP* Distal symmetric polyneuropathy (any form), *SSP* Supraspinatus tendon

**Table 5:** Associations between the echogenicity of the supraspinatus, infraspinatus, deltoid and biceps brachii muscle of the painful shoulders (expressed in z-scores) and clinical variables in patients without diabetes mellitus suffering from shoulder pain (*n =* 23)

Clinical variables	Muscles											
	Supraspinatus			Infraspinatus			Deltoid			Biceps brachii		
	B	95% CI	*P-*value	B	95% CI	*P-*value	B	95% CI	*P-*value	B	95% C	*P-*value
DSP	−0.8	−2.0, 0.3	0.148	− 0.7	− 2.0, 0.5	0.220	− 0.7	−3.0, 1.6	0.539	0.5	−1.2, 2.3	0.519
Signs of shoulder neuropathy	1.0	−0.2, 2.2	0.090	−0.4	−2.0, 1.2	0.599	−0.2	−3.2, 2.8	0.892	−1.1	−3.4, 1.1	0.302
Signs of cervical radiculopathy	0.01	−1.3, 1.3	0.978	0.2	−1.5, 2.0	0.768	0.04	−3.2, 3.3	0.978	0.7	−1.8, 3.1	0.571
Duration of pain	0.01	−0.01, 0.02	0.367	0.01	−0.01, 0.03	0.225	0.01	−0.02, 0.05	0.434	−0.01	−0.04, 0.01	0.408

B is multiple level linear regression coefficient; *DSP D*istal symmetric polyneuropathy (any form)

## Discussion

In this study, by using a non-invasive measure, we found that in painful shoulders of both patients with T2DM and patients without DM, the mean echogenicity z-score of the supraspinatus, infraspinatus, deltoid and biceps brachii was significantly increased compared to the reference values. No significant difference was found in muscle echogenicity between both patient groups, and in both groups approximately two-thirds of the painful shoulders had at least one muscle with an abnormal high z-scores (≥ 2.0), indicating pathology. Patients with T2DM tend to have more frequently abnormal high z-scores compared to patients without DM, but again, this was not statistically significant. This study also showed that a high prevalence of DSP in both patients groups, which was unexpected, as the estimated prevalence of DSP in the general Dutch population is about 4% [35].

In patients with T2DM, DSP was significantly associated with increased echogenicity z-scores of all muscles except for the infraspinatus muscle, while no statistically significant associations were observed in patients without DM.

These findings indicate that patients with painful shoulders seem to have abnormal muscles. The final common pathway that leads to shoulder pain, irrespective of having DM, is unknown, but might be muscle denervation. The observed increased echogenicity seems not attributed to disuse, commonly seen in patients with significant pain, as disuse will not lead to such significant muscle changes [36].

Any disorder both in the central and peripheral nervous system, including the muscle itself, can cause muscle denervation. This study did not include an assessment to diagnose underlying neurological disorders, except for a clinical assessment for cervical radiculopathy or classic neuralgic amyotrophy, which was present in both patient groups with almost equal percentages.

Interestingly, we observed no difference in mean z-scores of muscles echogenicity between painful shoulders and non-painful shoulders in patients with T2DM and without DM, indicating that also muscles in non-painful shoulders might be affected. Several explanations are possible for this observation. DM is associated with an increased intermuscular adipose tissue [21], leading to a higher echogenicity. Although we tried to match healthy participants within the BMI range of patients with T2DM, the mean BMI of the healthy participants was significantly lower. Although we corrected for it in our regression formulas, the influence of extreme high BMI on muscle echogenicity still cannot be excluded. High BMI (> 35) was present in 7.5% (*n =* 5) of the patients with T2DM, and in 4.3% (*n =* 1) of the patients without DM. Another explanation could be that in the group without DM, patients with undiagnosed DM were nevertheless included. We have not tested these patients if they have DM, but only asked if they were diagnosed with it. Finally, it could be that in muscles of asymptomatic shoulders, pathology had started, but the progression did not reach to symptomatic levels yet. It is known that half of diabetic neuropathies are asymptomatic [10].

Considering the results, we have to take into account some limitations. First, selection bias might be introduced by our recruitment strategies of patients with shoulder pain, which might hamper the generalizability of the results to the entire population of patients with shoulder pain. Patients with higher pain levels or a longer duration of pain might be more eager to participate, which might be an indication of more severe shoulder pathology. This might explain why the mean echogenicity is higher compared to healthy participants, and the majority of patients has at least one abnormal z-score. Moreover, the available timeslots in the diagnostic center were limited, resulting in a rather low sample of patients without DM. This might have led to a loss of statistical power. Second, we did not assess muscle thickness, an indicator of muscle atrophy, which is also a sign of muscle denervation [19, 37]. We excluded patients and healthy participants with a history of self-reported (poly-)neuropathies other than diabetic distal symmetric polyneuropathy, or myopathies, and did not assess them for these disorders. Finally, in order to better compare the study groups in the future, we recommend presenting the following additional clinical baseline variables: duration of shoulder pain, previous episodes of shoulder pain, medication (type of diabetes medication, statins etc)*.*

Our findings indicate that patients with painful shoulders, irrespective of having T2DM, seem to have abnormal shoulder muscles. We did not collect information on muscles outside the shoulder girdle to investigate the systematic effect of DM on other muscles, nor did we include muscle thickness measurements or nerve conducting studies. Future studies are needed to confirm these findings and further to elucidate whether neuropathy or other conditions lead to these muscle changes.

## Supplementary Information

**Additional file 1: Table S1.** Detailed results of the neurological feet and shoulder examination including established diagnosis in patients with type 2 diabetes mellitus and without diabetes mellitus suffering from shoulder pain. **Table S2.** Regression formulas for reference values of muscle echogenicity for females and males separately

## Data Availability

The datasets used and/or analysed during the current study are available from the corresponding author on reasonable request.

## Abbreviations

DM: Diabetes mellitus
DSP: Distal symmetrical polyneuropathy
QMUS: Quantitative muscle ultrasound
T2DM: Type 2 diabetes mellitus
