# Supplementary material for: Shoulder muscle changes in patients with type 2 diabetes mellitus who have a painful shoulder: a quantitative muscle ultrasound study
Source: BMC Musculoskelet Disord. 2022 Jul 14;23:674. doi: 10.1186/s12891-022-05627-9 (PMC9281102; doi:10.1186/s12891-022-05627-9)
Supplement: Supplementary file 1 — Additional file 1: Table S1. Detailed results of the neurological feet and shoulder examination including established diagnosis in patients with type 2 diabetes mellitus and without diabetes mellitus suffering from shoulder pain. Table S2. Regression formulas for reference values of muscle echogenicity for females and males separately [file 12891_2022_5627_MOESM1_ESM.docx]

**Supplementary data**

Table S1: Detailed results of the neurological feet and shoulder examination including established diagnosis in patients with type 2 diabetes mellitus and without diabetes mellitus suffering from shoulder pain

|  | **Patients suffering from shoulder pain** | |
| --- | --- | --- |
|  | **Type 2 diabetes** **mellitus**  **n=66 (%)** | **Without diabetes** **mellitus**  **n=23 (%)** |
| **Single signs of neurology feet examination**  Inspection  Hammertoe  Claw toe  Charcot foot  Any deformity | 1 (1.5)  11 (16.6)  2 (3.03)  13 (19.6) | 0  3 (13.0)  0  3 (13.0) |
| Abnormal ankle reflex | 50 (75.7) | 13 (56.5) |
| Distal muscle weakness | 6 (9.2) | 1 (4.3) |
| Abnormal vibration sense | 45 (69.2) | 18 (78.2) |
| Abnormal monofilament sense | 5 (7.5) | 0 |
| **Clinical DSP – presence of symptoms and combination of signs** |  |  |
| Presence of at least one neurological symptom**,** with decreased or absent ankle reflex, and decreased or absent distal sensation (vibration and/or monofilament) | 19 (28.8) | 4 (17.4) |
| Presence of at least one neurological symptom in combination with decreased or absent ankle reflex, and decreased or absent distal sensation (vibration and/or monofilament) with distal muscle weakness | 3 (4.5) | 1 (4.3) |
| **Subclinical DSP – presence of combination of signs only** |  |  |
| Combination of: decreased or absent ankle reflex, and decreased or absent distal sensation (Vibration and/or monofilament) | 37 (56.1) | 13 (56.5) |
| Combination of: decreased or absent ankle reflex, and decreased or absent distal sensation (Vibration and/or monofilament) with distal muscle weakness | 5 (7.6) | 1 (4.3) |
| **Neuropathic shoulder pain using DN4 questionnaire:**  Cutoff score≥4  Cutoff score≥5 | 7 (10.6)  2 (3.0) | 1 (4.3)  0 |

DSP : distal symmetric polyneuropathy ; DN4 : Douleur Neuropathique 4 questions.

Table S2: Regression formulas for reference values of muscle echogenicity for females and males separately

|  | Muscle echogenicity: EI= C + (α · age centered) + (β · age centered^2^) + (γ · age centered^3^) + (δ · BMI) + (ζ · dominant hand) | | | | | | | | | | | | | | | |
| --- | --- | --- | --- | --- | --- | --- | --- | --- | --- | --- | --- | --- | --- | --- | --- | --- |
| **Females** |  | | | | | | | | | | | | | | | |
| Muscle | Location | C | | α | | β | | γ | | δ | | ζ | | SD | | R^2^ |
| Supraspinatus  Right side | 1/4 along the line from the lateral acromion to the vertebral midline | Mean 47.006, SD 14.368 | | | | | | | | | | | | | | |
| Left side |  | Mean 45.496, SD 12.487 | | | | | | | | | | | | | | |
| Infraspinatus  Right side | Caudally from supraspinatus position, passing the scapular spine | 34.145 | | 0.411 | | 0 | | 0 | | 0 | | 0 | | 10.631 | | 0.154 |
| Left side |  | Mean 35.516, SD 8.665 | | | | | | | | | | | | | | |
| Deltoid  Right side | Middle deltoid muscle: 1/4 of the distance from the acromion to the lateral epicondyle | 96.066 | | 1.458 | | 0 | | -0.006 | | 0 | | -12.246 | | 8.895 | | 0.453 |
| Left side |  | 105.725 | | 1.896 | | 0 | | -0.007 | | -1.520 | |  | | 10.924 | | 0.445 |
| Biceps brachii  Right side | 2/3 of the distance from acromion to antecubital crease | Mean 74.289, SD 12.123 | | | | | | | | | | | | | | |
| Left side |  | 95.627 | 0.478 | |  | |  | |  | | -15.328 | | 13.278 | | 0.265 | |
| **Males** |  | | | | | | | | | | | | | | | |
|  | Location | C | | α | | β | | γ | | δ | | ζ | | SD | | R^2^ |
| Supraspinatus:  Right side | 1/4 along the line from the lateral acromion to the vertebral midline | Mean 35.313, SD 8.062 | | | | | | | | | | | | | | |
| Left side |  | Mean 35.404, SD 8.837 | | | | | | | | | | | | | | |
| Infraspinatus  Right side | Caudally from supraspinatus position, passing the scapular spine | 34.057 | | 1.026 | | 0 | | -0.003 | | 0 | | 0 | | 5.174 | | 0.538 |
| Left side |  | 30.151 | | 1.461 | | 0 | | -0.004 | | 0 | | 0 | | 6.350 | | 0.613 |
| Deltoid  Right side | Middle deltoid muscle: 1/4 of the distance from the acromion to the lateral epicondyle | 69.442 | | 0.835 | | 0 | | 0 | | 0 | | 0 | | 9.442 | | 0.369 |
| Left side |  | 63.883 | | 0.105 | | 0 | | 0.009 | | 0 | | 0 | | 8.998 | | 0.481 |
| Biceps brachii  Right side | 2/3 of the distance from acromion to antecubital crease | 68.179 | | 1.557 | | 0 | | -0.007 | | 0 | | 0 | | 11.656 | | 0.286 |
| Left side |  | 82.182 | | 0.794 | | 0 | | 0 | | 0 | | -8.683 | | 7.347 | | 0.676 |
